# South African speech-language therapists’ opinion of their training in cleft lip and palate and craniofacial deformities

**DOI:** 10.4102/sajcd.v67i1.695

**Published:** 2020-07-30

**Authors:** Emad Ghabrial, Kurt W. Bütow, Steve A.S. Olorunju

**Affiliations:** 1 Department of Orthodontics, Faculty of Health Sciences, University of Pretoria, Pretoria, South Africa; 2 Division of Dentistry, Faculty of Health Sciences, University of KwaZulu-Natal, Durban, South Africa; 3 Biostatistics Unit South African Medical Research Council, Pretoria, South Africa

**Keywords:** speech-language therapist, cleft palate, cleft lip, multidisciplinary, education, professional development, medical survey, craniofacial disorder

## Abstract

**Background:**

Speech care of cleft lip and/or palate (CLP) and craniofacial deformities (CFD) is complex and lengthy and requires collaboration amongst different disciplines. Consequently, it is important to provide academic educational models that include didactics, online learning and clinical exposure in CLP and CFD treatment, and participation in established cleft palate multidisciplinary team management.

**Objectives:**

To obtain information regarding: (1) the perceived adequacy of CLP and CFD academic education of speech-language therapists (SLTs); (2) the professional services that SLTs offer to CLP and CFD patients; and (3) the educational needs of SLTs in this field.

**Method:**

A 54-item online survey to collect quantitative data was conducted by telephone and email using a randomised sample of SLTs in different areas of South Africa.

**Results:**

The questionnaire was completed by 123 SLTs, 70% of whom had more than 10 years of professional experience. Of the respondents, 81% acknowledged their limited clinical exposure during their academic education. Only 42% of the professionals offer treatment for CLP and CFD patients. Of the respondents, 96% agreed on the need to improve CLP and CFD academic education, and the majority recommended certified courses, continued-education workshops and online resources.

**Conclusion:**

The findings indicate that SLTs academic training is perceived to be significantly limited in the cleft palate and craniofacial fields. Thus, there is a strong need at the undergraduate level for clinical training and exposure to multidisciplinary management. At post-graduate level there is a need to establish an educational strategy to meet the needs of SLTs providing CLP and CFD care. Participants suggested that programmes for continuing professional education, degree courses and online resources be designed to provide practising clinicians with updated information and guidance in management of CLP and CFD patients.

## Introduction

Speech therapy is considered a core service in the management of cleft lip and/or palate (CLP) and craniofacial deformities (CFD), since young children with CLP are at considerable risk of suffering from delayed or disordered communication development (American Cleft Palate-Craniofacial Association, 2016; Hammond & Stassen, 1999). The role of the speech-language therapist (SLT) dealing with children with CLP and/or CFD is essential, not only to achieve the maximum communication potential but also in the management of swallowing and feeding (Evens & Louw, 2015; Fair & Louw, 1998; Peterson-Falzone,Trost-Cardamone, Karnell & Hardin-Jones, 2016).

For many years, researchers and practitioners have understood the need to enhance academic education and clinical experience regarding CLP and CFD in order to provide quality management and to improve access to care for all patients and their families (Dabed & Cauvi, 1998; Gadbury-Amyot, Simmer-Beck, McCunniff & Williams, 2006). One of the earliest attempts to evaluate academic education in the CLP field was made by Lass, Gasperini, Overberger and Connolly (1973), to assess the exposure of academic students to CLP treatment by means of a questionnaire.

The main finding was that there was a lack of clinical exposure and basic theoretic education. This was the same as the findings of Vallino, Lass, Pannbacker, Klaiman and Miller (1992) on the effects of limited academic training in CLP management; they advise that a clinician with limited training should not manage individuals with CLP.

CLP patient care relies on the teaching and exposure that the student received at university and the knowledge gained throughout the practitioner’s career (McDonald, Adamidis, Eaton, Seeholzer & Sieminska-Piekarczyk, 2000; Wium & Louw, 2013). Therefore, continuous evaluation of the academic education and services provided for CLP and CFD is necessary to provide a foundation for the improvement of educational resources.

It becomes necessary to obtain information regarding the current knowledge of SLTs in the CLP field and also to determine the educational needs of those providing services to CLP and CFD (Cameron & Widmer, 2013).

## Methods

Ethical permission was obtained from the Humanities and Social Sciences Research Ethics Committee of the University of KwaZulu-Natal. A quantitative research method using a 54-item structured questionnaire was developed. Qualtrics Research Suite survey software (Qualtrics, 2017) was used to capture and analyse the data. The questionnaire was designed to collect quantitative data using a Likert-type scale, which was introduced to each practitioner by means of a telephone call. Consent for participation was obtained from each respondent prior to completing the questionnaire. The data was collected either online or in a telephone interview, according to the preference of the participants.

## Questionnaire design

The questionnaire consisted of four sections: the first determined whether the participants were acceptable for inclusion in the study. The second section collected the level of knowledge, experience and services provided by the participants. In the third section, their educational needs and preferences regarding further education were determined. The last section collected demographic data, which included title, gender, age, degree(s) and location by region.

## Selection of participants

A random sample of SLTs was obtained from the Medpages database registry for practising healthcare professionals (Manana et al., 2018). Regarding sample size, the author used the literature information (Modi & Ross, 2000; Thandeka, Penelope & Robin, 2016) as a guidance for the response rate. This was reviewed upward to 19% of Medpages’ practising SLTs to account for a possible sampling error of 15%.

## Distribution

The researcher approached the South African Speech-Language-Hearing Association to distribute the survey by email. Before distribution, the questionnaire was piloted by selected practitioners. They were invited to complete the questionnaire, which was subsequently revised based on the responses, in order to ensure the appropriate capturing of data. Initially, the questionnaires were to be distributed by the Qualtrics online survey platform twice during the first week, then weekly afterwards. This was ultimately not necessary, as the targeted participant number was achieved by randomly contacting 123 SLTs on the Medpages database.

## Data analysis

The data were captured using Excel 2013. This was later converted into Stata 15 s (string) format. The analysis undertaken was descriptive summary statistics presenting frequencies and associated percentages. No further analytical tools were used because no hypothesis was being tested.

### Ethical consideration

This article followed all ethical standards for carrying out research with ethical clearance obtained from University of Kwazulu-Natal, School of Health Sciences.

## Results

**FIGURE 1:**
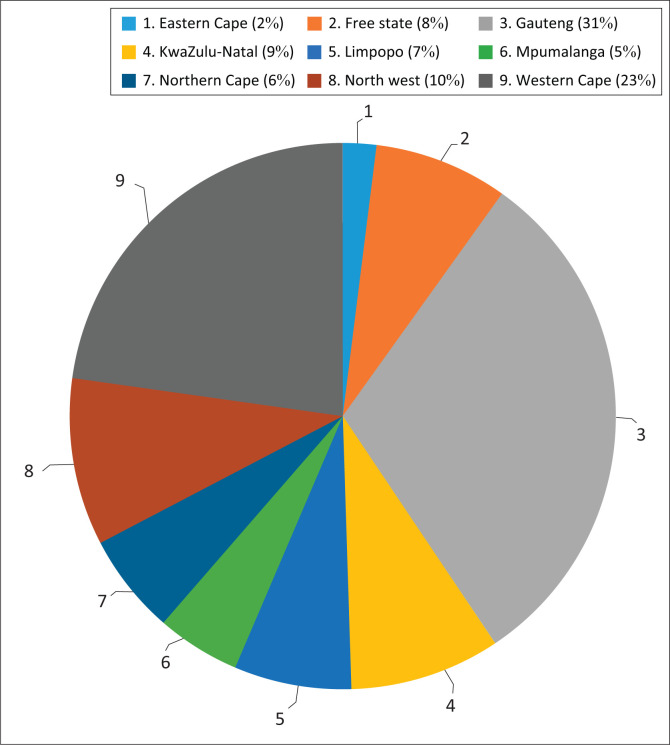
Respondents’ distribution according to province.

**FIGURE 2:**
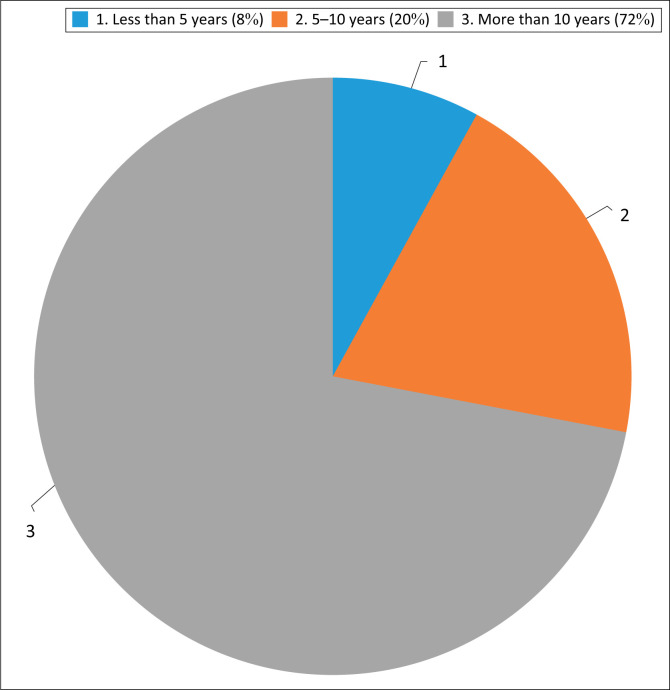
Respondents’ distribution according to clinical experience.

The questionnaire was completed by 123 SLTs, representing most of South Africa’s provinces (Figure 1). Of these practitioners, 70% had more than 10 years of professional experience (Figure 2).

Regarding the basic knowledge questions, 39% of the respondents were uncertain of the correct answers. These questions were:

Q8. The most common craniofacial facial deformity in humans is cleft lip/palate. True/False/Do not know.

Q9. The incidence of cleft lip/palate is higher in black populations than in white population. True/False/Do not know.

When asked about their educational experience, only 9% stated that they had clinical exposure during their undergraduate education and 13% during postgraduate education. Only 8% at undergraduate level and 13% at postgraduate level participated in multidisciplinary meetings during their academic education.

**FIGURE 3:**
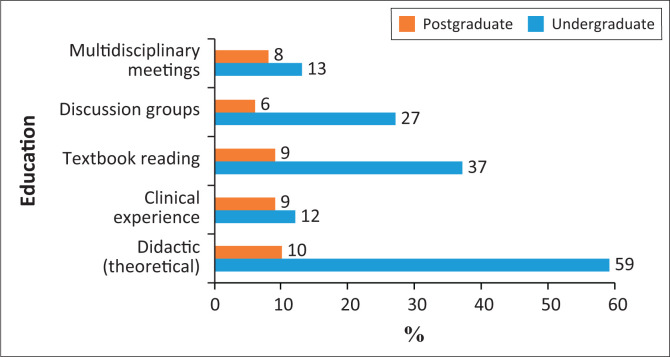
Undergraduate and postgraduate academic education in cleft lip/palate/craniofacial deformities patient management.

Regarding didactic exposure, only 10% of the undergraduates and 59% of the postgraduates had some exposure (Figure 3). Just 44% of the respondents offered services for both CLP and CFD patients and 41% of them participated in multidisciplinary teams. Regarding the services provided according to patients’ age groups, only 10 of the participants offer assessment and intervention to infants. The rest offer services to children from the age of 4 years to adult. When the respondents were asked about the facility where CLP patients were consulted and treated, the following emerged: private practice 47%, private hospitals 24% and academic and/or government hospitals 25%. Only three of the respondents offered their services at special schools and government clinics.

The 53 respondents who stated that they offer assessment, as well as treatment were asked about the complexity of CLP/CFD patients. They acknowledged that it is exceptionally difficult to treat these patients and that it requires special training.

When asked why there is a need for special training, they indicated that it is due to the multidisciplinary approach needed (35%), the lengthy treatment (34%), and patients’ socio-economic situation (25%). In the open-ended questions, a few respondents cited the emotional state of the family and patients as being additional complicating factors.

**FIGURE 4:**
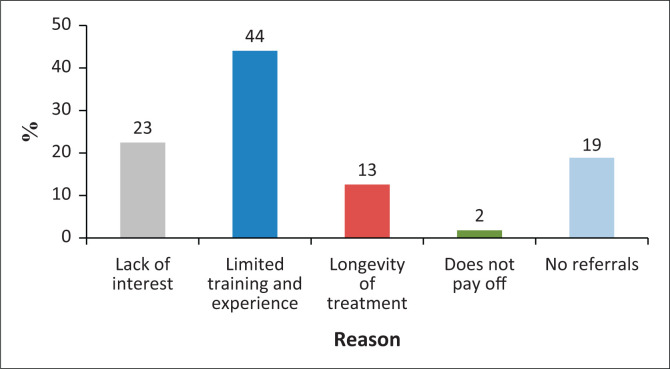
What prevents speech-language therapists from treating cleft lip/palate/craniofacial deformities patients?

When the non-treating respondents were asked to highlight the factors preventing them from treating CLP and CFD patients, 44% stated that it was related to limited training and experience, 23% admitted lack of interest, 19% stated that it was because of low referrals and 13% highlighted the duration of the treatment (Figure 4).

Almost all of the respondents agreed on the need to improve the academic education offered to CLP and CFD treatment providers, and 97% of the respondents recommended dedicated academic training programmes in that field. Of the respondents, 62% would like to further their knowledge of CLP and CFD management.

**FIGURE 5:**
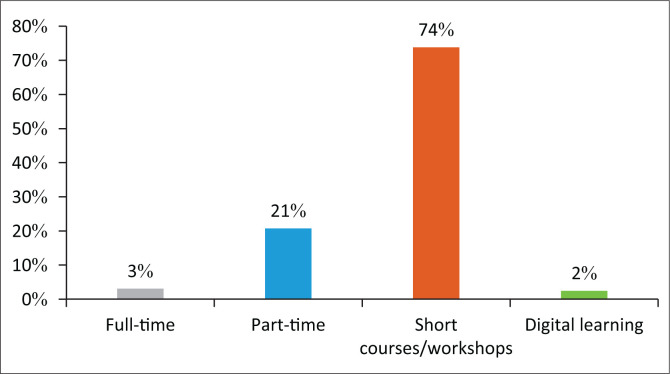
Programme for cleft lip/palate/craniofacial deformities format.

When participants were asked about the preferred method of education:

- the majority 74% recommended short courses and workshops.
- 21% recommended part-time certificate courses.
- 3% recommended full-time studies; and
- only 2% recommended digital learning (Figure 5).

**FIGURE 6:**
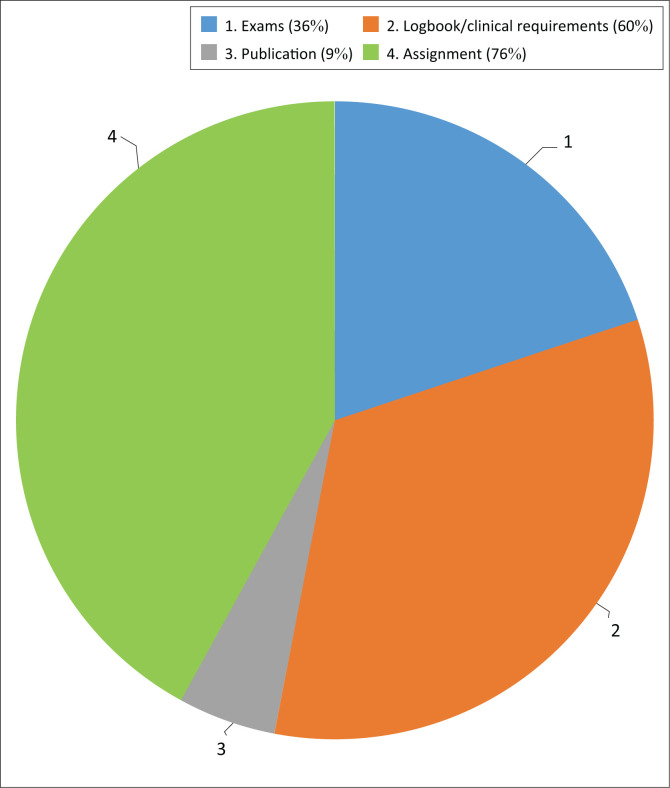
What form of evaluation would you suggest?

Participants identified interest, passion and the prospect of joining an interdisciplinary team as the most significant reasons for enrolment in CLP and CFD-dedicated courses. When asked about a certificate course, the participants suggested that the focus should be on assessment, treatment planning, clinical skills, and an interdisciplinary approach. Keeping a logbook of the hours spent in clinical training, as well as participating in examinations, were generally recommended as effective evaluation methods for certificate courses (Figure 6).

## Discussion

As indicated by Callahan and Hazelwood (2004), as the speech-language therapy field widens the less-frequently seen CLP and CFD patients present a challenge for academic training and practitioners because of less emphasis on academic education and changes in the scope of practice in some countries. In this survey, the majority of the respondents received an academic education that included mandatory course work. This is in line with the findings of Vallino, Lass, Bunnell and Pannbacker (2008). Despite this, respondents expressed the need for further training and clinical exposure to make them competent to provide services to CLP and CFD patients.

## Survey distribution

Some previous SLT surveys, e.g. Pannbacker, Lass, Scheuerle and English (1992), used email or postal questionnaires. Others used incentives to improve the response rate (Bedwinek, Kummer, Rice & Grames, 2010). This study used a mixed method of data collection: telephone interviews and email provided wide distribution not limited by email access, in line with recommendations by Flanigan, McFarlane and Cook (2008). By targeting a number of responses to obtain a statistically valid number, it was possible to reach the required number with less concern about a low response rate than that experienced by Asch, Jedrziewski and Christakis (1997).

## Sample

In similar studies, Pannbacker et al. (1992) surveyed SLTs who were members of the American Cleft Palate Association. The sample in the study by Bedwinek et al. (2010) comprised SLTs from selected schools. These surveys provided valuable information but did not include any other types of SLT. In this study, attention has been given to including information obtained from SLTs with varied years of experience, from different locations and places of employment, in order to overcome limitations and to obtain general opinions from all clinicians.

## Academic education

This study found a limited emphasis on general knowledge and clinical exposure during academic graduate programmes. This is in line with the findings of Callahan and Hazelwood (2004) and Kuehn, Kummer, D’Antonio and Karnell (2006) that graduate students may leave with limited education in the CLP fields. This study found similar data on academic education with limited clinical and multidisciplinary exposure. The findings are in line with those of Pannbacker, Lass and Starr (1979), demonstrating that practitioners who are legally qualified to provide treatment really know very little about these deformities.

## Educational needs and strategy

This investigation revealed a strong desire amongst SLTs for professional development and dedicated educational programmes in the areas of assessment and intervention with children born with CLP and CFD, in line with the findings of Vallino et al. (2008). Most respondents preferred practical information and multidisciplinary exposure related to CLP and CFD patients. Of the respondents, 73% ranked continued professional development as a preferred way of obtaining information, and 32% recommended a certification programme of 12–24 months. This contrasts with the finding of Bedwinek et al. (2010) that web-based education and conferences are the preferred method of continuing education.

## Conclusion

As the SLT field widens, it leads to less education in uncommon problems such as CLP and CFD. Speech-language therapists may be confronted with the need to provide services that are not covered by their training and experience. It is essential for SLTs to know how to assist the patient and communicate with the appropriately-trained professionals. This study shows that there is a demand from SLT practitioners for continued education and certificate courses in the CLP field. Consequently, academic institutions need to adopt educational strategies and provide resources for under- and postgraduate students and practitioners. Nevertheless, knowledge exchange through online communities will benefit a wider range of SLTs (Karnell, Bailey, Johnson, Dragan & Canady, 2005).

### Limitations

This survey represents the opinions of those SLTs who were selected randomly and who were willing to respond to the survey. It is possible that those SLTs who received the survey and did not respond, did not feel that additional training in this area was a need or concern. Another limitation is the lack of verification of the actual training in CLP and CFD by the universities, to justify whether the perceptions are valid or not, which could raise a whole new set of questions.
